# Products of Oxidative Guanine Damage Form Base Pairs with Guanine

**DOI:** 10.3390/ijms21207645

**Published:** 2020-10-15

**Authors:** Katsuhito Kino, Taishu Kawada, Masayo Hirao-Suzuki, Masayuki Morikawa, Hiroshi Miyazawa

**Affiliations:** 1 Kagawa School of Pharmaceutical Sciences, Tokushima Bunri University, 1314-1 Shido, Sanuki, Kagawa 769-2193, Japan; s108019@stu.bunri-u.ac.jp (T.K.); mmorikawa@kph.bunri-u.ac.jp (M.M.); miyazawah@kph.bunri-u.ac.jp (H.M.); 2 Laboratory of Xenobiotic Metabolism and Environmental Toxicology, Faculty of Pharmaceutical Sciences, Hiroshima International University (HIU), 5-1-1 Hiro-koshingai, Kure, Hiroshima 737-0112, Japan; m-hirao@hirokoku-u.ac.jp

**Keywords:** oxidative guanine damage, base incorporation, base pair, G-C transversions, 2019 novel coronavirus, SARS-CoV-2

## Abstract

Among the natural bases, guanine is the most oxidizable base. The damage caused by oxidation of guanine, commonly referred to as oxidative guanine damage, results in the formation of several products, including 2,5-diamino-4*H*-imidazol-4-one (Iz), 2,2,4-triamino-5(2*H*)-oxazolone (Oz), guanidinoformimine (Gf), guanidinohydantoin/iminoallantoin (Gh/Ia), spiroiminodihydantoin (Sp), 5-carboxamido-5-formamido-2-iminohydantoin (2Ih), urea (Ua), 5-guanidino-4-nitroimidazole (NI), spirodi(iminohydantoin) (5-Si and 8-Si), triazine, the M+7 product, other products by peroxynitrite, alkylated guanines, and 8,5′-cyclo-2′-deoxyguanosine (cG). Herein, we summarize the present knowledge about base pairs containing the products of oxidative guanine damage and guanine. Of these products, Iz is involved in G-C transversions. Oz, Gh/Ia, and Sp form preferably Oz:G, Gh/Ia:G, and Sp:G base pairs in some cases. An involvement of Gf, 2Ih, Ua, 5-Si, 8-Si, triazine, the M+7 product, and 4-hydroxy-2,5-dioxo-imidazolidine-4-carboxylic acid (HICA) in G-C transversions requires further experiments. In addition, we describe base pairs that target the RNA-dependent RNA polymerase (RdRp) of RNA viruses and describe implications for the 2019 novel coronavirus (SARS-CoV-2): When products of oxidative guanine damage are adapted for the ribonucleoside analogs, mimics of oxidative guanine damages, which can form base pairs, may become antiviral agents for SARS-CoV-2.

## 1. Introduction

The DNA bases guanine, adenine, thymine, and cytosine have oxidation potentials (vs. NHE) of 1.29, 1.42, 1.6, and 1.7 V, respectively, and among them, guanine is most susceptible to oxidation [1]. Oxidation of guanine triggers mutations. Indeed, mutations resulting from a change from guanine to other bases have been detected in the oncogene *K-ras* and the tumor suppressor gene *p53* [2,3,4]. G-T transversions and G-C transversions are preferentially caused by sunlight, ultraviolet light in the presence of riboflavin or menadione, visible light with methylene blue, Fe^2+^, hydrogen peroxide, peroxy radicals, endoperoxide, dioxetane, γ irradiation, and smoking [5], and all these processes are induced by oxidative stress. The products of oxidative guanine damage that can pair with adenine cause G-T transversions. Alternatively, those that can pair with guanine cause G-C transversions.

8-Oxoguanine (8-oxoG), which is known as oxidative guanine damage, causes G-T transversions by pairing with adenine [6,7]. Many products of oxidative guanine damage cause G-T transversions, but few cause G-C transversions [8,9,10]. Actually, adenine is predominantly incorporated opposite an apurinic/apyrimidinic site; this “A rule” suggests that the insertion of adenine may not depend on the formation of hydrogen bonds with the DNA damage [11]. Conversely, when guanine is preferentially incorporated, hydrogen bonds certainly form between the DNA damage and guanine [12]. Therefore, base pairs containing guanine damages with guanine are highlighted in this review, and these base pairs can lead to G-C transversions. In addition, we pick up base pairs containing antiviral drugs, which target the RNA-dependent RNA polymerase of RNA viruses and describe implications involving oxidative guanine damage for the 2019 novel coronavirus (SARS-CoV-2).

## 2. 2,5-Diamino-4*H*-imidazol-4-one (Iz) and 2,2,4-triamino-5(2*H*)-oxazolone (Oz)

**Figure 1:**
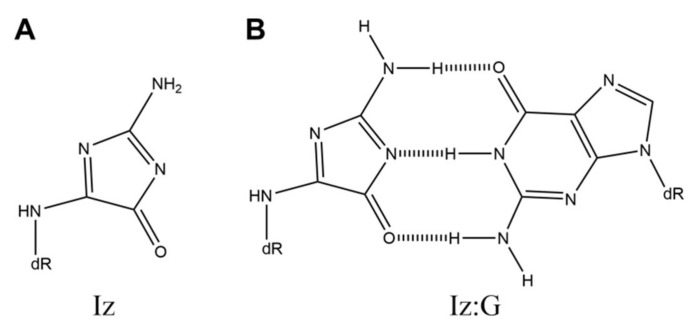
The structures of (**A**) Iz and (**B**) Iz:G.

2,5-Diamino-4*H*-imidazol-4-one (Iz) (Figure 1A) is a product of oxidative guanine damage. Iz can be produced by the oxidation of guanine with Mn-TMPyP/KHSO_5_, photooxidation in the presence of riboflavin or anthraquinone, or oxidation by γ radiation [13,14,15,16]. Iz can also be produced by the oxidation of guanine and 8-oxoG with peroxynitrite [17] or by photooxidation of 8-oxoG in the presence of riboflavin and methylene blue under basic conditions [14,18,19].

The structure of Iz can form a base pair with guanine (Figure 1B). In 1998, their *ab initio* molecular orbital calculations of the base pair containing Iz indicated that the Iz:G base pair is predicted to be stable [15]. Other calculations for the Iz:G base pair have also been confirmed [20]. In addition, Iz:G base pairs are the most thermally stable because the *T*_m_ values of base pairs are as follows: Iz:G, 37.1 °C; Iz:T, 27.3 °C; Iz:A, 24.9 °C; Iz:C, 23.7 °C [5]. In 2001, base incorporation studies were performed using *Escherichia coli* (*E. coli*) DNA polymerase I. The results of these studies show that guanine is dominantly incorporated opposite Iz, and that the Iz triphosphate is only incorporated opposite guanine [14]. It is also suggested that Iz forms a base pair with guanine [21]. In addition, guanine is incorporated opposite Iz when human DNA polymerase α or rat DNA polymerase β is used [22]. Furthermore, human DNA polymerase η incorporates guanine in addition to cytosine, adenine, and thymine [23]. Thus, all of the DNA polymerases incorporate guanine opposite Iz. Furthermore, when replicated *in vivo* in *E. coli*, Iz induces mainly G-C transversions [24].

**Figure 2:**
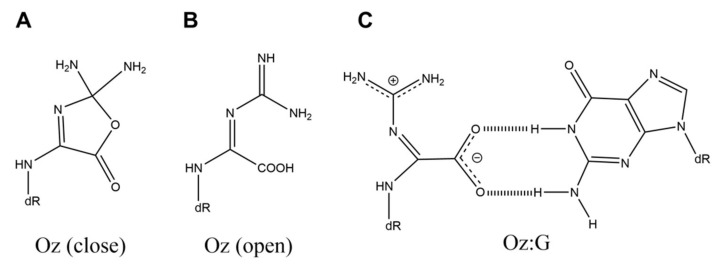
The structures of (**A**) Oz (the closed-ring structure), (**B**) Oz (the open-ring structure), and (**C**) Oz:G.

Based on these experimental results, Iz can be considered to be a product of oxidative guanine damage that can cause G-C transversions. However, Iz has low thermal stability (half-life of 2.5 h at 37 °C [16]) and decomposes into 2,2,4-triamino-5(2*H*)-oxazolone (Oz) (Figure 2A). Oz has been detected in rat liver DNA [25], and this report shows that Iz is degraded under the near-neutral condition in rat liver.

The large fragment of *E. coli* DNA polymerase I exonuclease minus (Klenow fragment exo^-^) incorporates adenine opposite Oz, and Taq DNA polymerase mostly incorporates adenine opposite Oz [26]. However, when base incorporation opposite Oz using human DNA polymerase α was first analyzed, their findings reveal that guanine, rather than adenine, is predominantly incorporated [22].

Oz has been reported to have a closed ring structure [26], which does not appear to be able to form base pairs with guanine. However, since the closed-ring structure of Oz has an ester structure, the ring can be opened by hydrolysis, forming an open-ring structure. It has been noticed that the open-ring structure of Oz (Figure 2B) could form two hydrogen bonds with guanine. *Ab initio* calculation suggests the existence of the base pair structure shown in Figure 2C [22]. Moreover, unlike the closed-ring structure of Oz, the open-ring structure of Oz can be planar, contributing to the stabilization of stacking and conferring an advantage in the DNA elongation reaction because a planar structure has less steric hindrance. In addition, we note that the *T*_m_ values of base pairs are as follows: G:C, 55.1 °C; T:A, 48.9 °C; Oz:G, 45.7 °C [27]. A G:C base pair has three hydrogen bonds, and a T:A base pair has two. Since the *T*_m_ value of an Oz:G base pair is close to that of T:A, Oz:G base pairs are thought to have two hydrogen bonds (Figure 2C) [27]. Other calculations also show that there are two hydrogen bonds in Oz:G base pairs [20].

In addition to human DNA polymerase α, guanine is incorporated opposite Oz by calf thymus DNA polymerase α, human DNA polymerase β, rat DNA polymerase β, human DNA polymerase δ, and yeast DNA polymerase ε [22,28]. *Sulfolobus solfataricus* DNA polymerase IV, human DNA polymerase γ, and human DNA polymerase κ incorporate guanine and adenine opposite Oz [22,28]. Human DNA polymerase ι and yeast DNA polymerase ζ incorporate guanine, cytosine, adenine, and thymine opposite Oz [28]. Human DNA polymerase η incorporates guanine, adenine, and cytosine opposite Oz [23]. Thus, for all of the polymerases mentioned above, guanine is either the base or is among the bases that could be incorporated opposite Oz.

In *E. coli*, G-T transversions are predominant in the mutation spectrum analysis, whereas G-C transversions are barely detected [29]. As mentioned earlier, adenine is predominantly incorporated opposite Oz [26]. The difference between these results [26] and the subsequent results [22,27] may be due to sequence-dependent effects. The possibility of sequence-dependent effects on base incorporation by polymerases should be examined in the future.

Based on these findings, we propose that in addition to Iz, Oz might also cause G-C transversions.

## 3. Guanidinoformimine (Gf)

**Figure 3:**
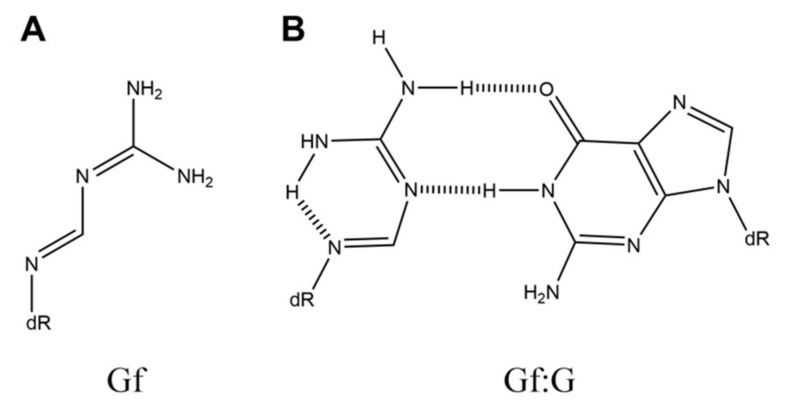
The structures of (**A**) Gf and (**B**) Gf:G.

Guanidinoformimine (Gf) (Figure 3A) can be produced by decarboxylation of Oz [30]. Base incorporation opposite Gf has been analyzed using the pyrosequencing method with Klenow fragment exo^-^, human DNA polymerase κ, and yeast DNA polymerase η [30]. In the case of Klenow fragment exo^-^, cytosine, adenine, and guanine are incorporated opposite Gf. Human DNA polymerase κ incorporates guanine, cytosine, and adenine opposite Gf. When yeast DNA polymerase η is used, guanine and cytosine are incorporated opposite Gf. As a result of base incorporation, Gf is predicted to be able to form a Gf:G base pair with two hydrogen bonds (Figure 3B) [30].

## 4. Guanidinohydantoin/Iminoallantoin (Gh/Ia) and Spiroiminodihydantoin (Sp)

**Figure 4:**
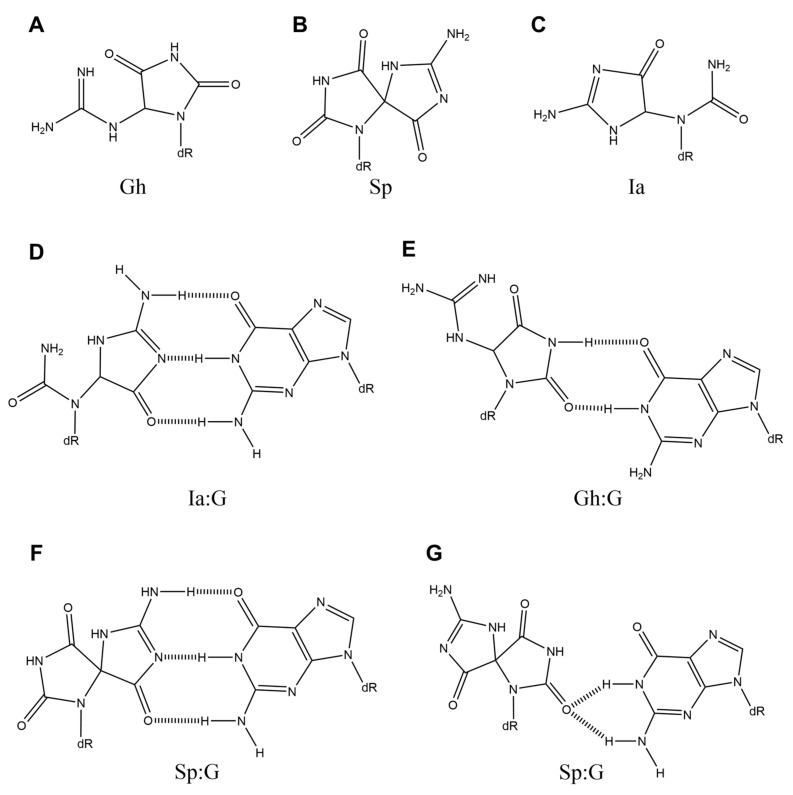
The structures of (**A**) Gh, (**B**) Sp, (**C**) Ia, (**D**) Ia:G, (**E**) Gh:G, and (**F** and **G**) Sp:G.

Using reaction mixtures of 8-oxoG with Na_2_IrCl_6_ without isolating each lesion as templates, guanine is incorporated in addition to adenine opposite the products by Klenow fragment exo^-^ [31]. No base is incorporated by calf thymus DNA polymerase α or human DNA polymerase β [31]. Two products are found: One product has a mass corresponded to 10 amu (M-10) below that of 8-oxoG. The other product has a mass corresponded to 16 amu (M+16) above that of 8-oxoG. The authors noted that the M-10 product is guanidinohydantoin (Gh) (Figure 4A), and the M+16 product is 5-OH-8-oxoG [31]. However, a year later, these authors reidentified 5-OH-8-oxoG as spiroiminodihydantoin (Sp) (Figure 4B) [32]. Furthermore, 4-OH-8-oxoG, as reported by Ravanat et al. [33], is actually Sp [34]. After that, Gh and Sp are separated [35].

In this paragraph, we discuss Gh. Klenow fragment exo^-^ incorporates guanine and adenine opposite Gh, and the *k*_cat_/*K*_m_ for the incorporation of adenine is less than that for guanine [36]. However, Gh:A base pairs are more thermally stable than Gh:G base pairs because the *T*_m_ values of Gh:A base pairs are higher than those of Gh:G base pairs [36]. The efficiency of incorporation of nucleotides opposite Gh is influenced by adjacent bases [36]. Moreover, the stabilities of Gh:G and Gh:A seem to depend on their positions in the sequence [37].

Gh is in equilibrium with iminoallantoin (Ia) (Figure 4C) [38], and, in 2005, it was described that Ia could form a base pair with guanine as Ia has the same structural moiety as Iz (Figure 4D) [5]. Subsequently, a Gh:G base pair (Figure 4E), which is based on the G:T wobble base pair, was proposed in 2007 [39]. *Ab initio* calculations for the base pairs shown in Figure 4D,E have indicated that the energy of the base pair shown in Figure 4D is more stable [40]. This result was confirmed by Jena et al. [41,42]. Indeed, in further experiments, human DNA polymerase α [22], calf thymus DNA polymerase α [22], human DNA polymerase β [22], rat DNA polymerase β [22], human DNA polymerase γ [22], yeast DNA polymerase ε [22], human DNA polymerase η [23], Klenow fragment exo^-^ [22,36], *Sulfolobus solfataricus* DNA polymerase IV [22], and bacteriophage DNA polymerase RB69 exo^-^ [43,44] commonly incorporated guanine opposite Gh/Ia. During transcription by yeast RNA polymerase II, although adenine is predominantly incorporated opposite Gh/Ia, guanine is also incorporated [45]. In reverse transcription by SuperScript III, guanine is incorporated opposite Gh/Ia, in addition to adenine [46]. Furthermore, mutational spectrum analysis in *E. coli* [47,48,49] yields results that suggest the presence of Gh/Ia:G base pairs.

In 2016, it was reported that Gh dominates at pH < 10.1 and Ia at pH > 10.1 [50]. Since the amount of guanine insertion by Klenow fragment exo^-^ increases at higher pH [50], Ia, but not Gh, can form base pairs with guanine and causes G-C transversions.

Finally, we discuss Sp. After Gh/Ia and Sp are separated [35], the incorporation reaction of Klenow fragment exo^-^ with Sp has been analyzed [36]. As a result, guanine is incorporated in addition to adenine. The *T*_m_ values of Sp:A and Sp:G base pairs differ depending on the surrounding sequence [36]. Similarly, the result of experiments of T4 DNA ligation suggests that the stabilities of Sp:G and Sp:A depend on their position in the sequence [37].

Like Ia, Sp has the same structural moiety as Iz, and Sp can form a base pair with guanine that has three hydrogen bonds (Figure 4F) [5]. Base pairs of Sp and guanine have been calculated, and the optimized structures have been obtained [40]. Similar calculations are also reported [51]. In addition, another Sp:G base pair (Figure 4G) has been proposed [52], and it is different from the base pair shown in Figure 4F.

Sp has a chiral carbon that can be separated by HPLC as two diastereomers, Sp1 and Sp2. Although the absolute stereochemistry remains to be determined, analyses of the differences in thermal stability and base incorporation of Sp1 and Sp2 have been performed, and the results are as follows. When the *T*_m_ values of both Sp1 and Sp2 are measured, the thermal stability of a base pair with guanine is found to be higher than that with adenine [53]. Mutation spectrum analysis in *E. coli* [47,48,49] suggests that guanine forms base pairs with both Sp1 and Sp2. DNA polymerase V in *E. coli* increases G-C transversions in the Sp2 site [54] and then is thought to incorporate guanine opposite Sp2.

In 2009, the absolute configurations of two stereoisomers, Sp1 and Sp2, were determined: Sp1 is in the (−)-*S* configuration, and Sp2 is in the (+)-*R* configuration [55]. Regarding *S*-Sp and *R*-Sp, Klenow fragment exo^-^, *Sulfolobus solfataricus* DNA polymerase IV, and hemo KlenTaq DNA polymerase incorporate guanine opposite *S*-Sp and *R*-Sp in addition to adenine [56]. However, *S*-Sp and *R*-Sp triphosphates are incorporated only opposite cytosine, indicating that Sp:G base pairs are not formed when *S*-Sp and *R*-Sp triphosphates are used [57]. In a transcription reaction with yeast RNA polymerase II, adenine is incorporated opposite *S*-Sp and *R*-Sp more often than guanine [45]. Besides, SuperScript III incorporates guanine and adenine opposite both *S*-Sp and *R*-Sp [46].

Based on these findings, Ia and Sp are oxidative guanine damages, which are involved in the generation of G-C transversions.

## 5. 5-Carboxamido-5-formamido-2-iminohydantoin (2Ih)

**Figure 5:**
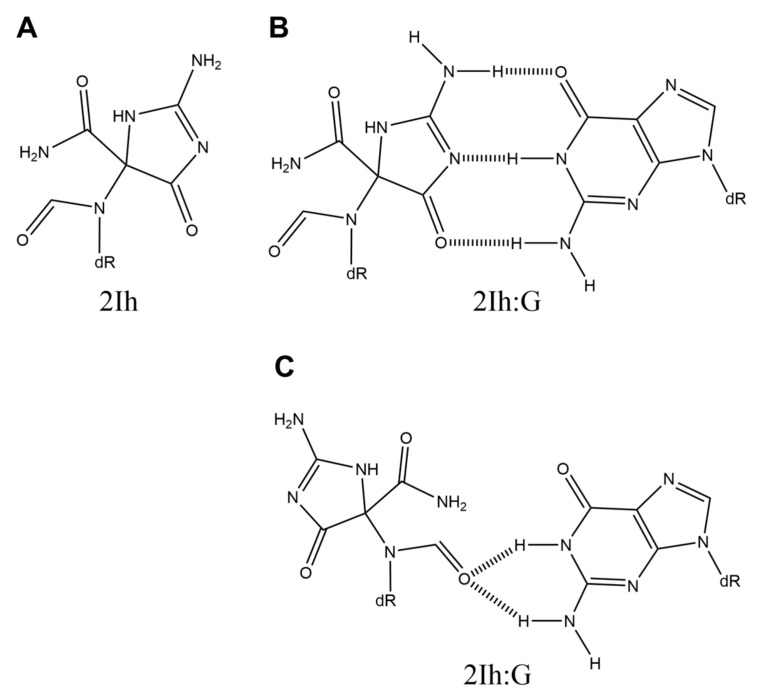
The structures of (**A**) 2Ih and (**B** and **C**) 2Ih:G.

5-Carboxamido-5-formamido-2-iminohydantoin (2Ih) (Figure 5A) is produced from guanine by Fe(II) or Cu(II) under reducing conditions, X-ray irradiation, or by manganese or nickel complex with KHSO_5_ [58,59,60,61,62].

2Ih has a chiral carbon, which can be separated by HPLC as two diastereomers. The thermal stability and base incorporation of *S*-2Ih and *R*-2Ih have been analyzed. The results of *T*_m_ analyses reveal that both *S*-2Ih and *R*-2Ih have higher thermal stability when base-paired with guanine than they do when base-paired with adenine [56]. Because the structure of 2Ih is similar to that of Sp, the 2Ih:G base pairs appear to be the structures shown in Figure 5B,C. In addition, analyses of base incorporation using Klenow fragment exo^-^, *Sulfolobus solfataricus* DNA polymerase IV, and hemo KlenTaq DNA polymerase show that guanine is predominantly incorporated opposite both *S*-2Ih and *R*-2Ih [56].

Although there has thus far been only one report of these findings, 2Ih is a candidate for a product of oxidative guanine damage that can cause G-C transversions.

## 6. Urea (Ua)

**Figure 6:**
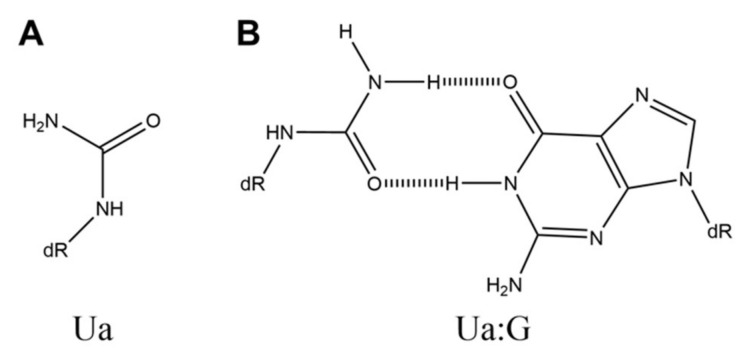
The structures of (**A**) Ua and (**B**) Ua:G.

Urea (Ua) is generated from thymine by osmium tetroxide (Figure 6A) [63] and has been reported to be a product of oxidative guanine damage [64].

Molecular mechanics calculations have proposed that the Ua:G base pair has one hydrogen bond [65]. However, the Ua:G base pair was previously proposed to have two hydrogen bonds (Figure 6B) [66]. Analysis of the mutation spectrum in *E. coli* [48,64,65] also suggests that Ua:G base pairs are formed. DNA polymerase V in *E. coli* increases G-C transversions in the Ua site [54] and then is thought to incorporate guanine opposite Ua.

## 7. 5-Guanidino-4-nitroimidazole (NI)

**Figure 7:**
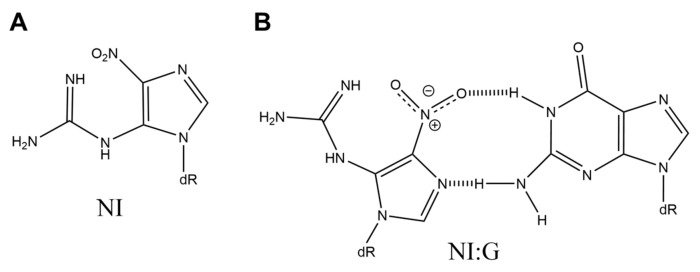
The structures of (**A**) NI and (**B**) NI:G.

In 2001, it was reported that 5-guanidino-4-nitroimidazole (NI) (Figure 7A) is produced by the oxidation of guanine by peroxynitrite [17]. NI is also generated by photooxidation of guanine with NaHCO_3_, NaNO_2_, and Na_2_S_2_O_8_ [67]. The *T*_m_ values of NI:G base pairs are higher than those of NI:A, NI:C, and NI:T base pairs [68], and the *ab initio* calculations for the base pairs have indicated that NI:G base pairs, shown in Figure 7B, are stable [69]. Calf thymus DNA polymerase α incorporates guanine and adenine opposite NI [70]. However, cytosine is predominantly incorporated opposite NI by human DNA polymerase β [70], Klenow fragment exo^-^ [70], human RNA polymerase II [71], and bacteriophage T7 RNA polymerase [71]. Furthermore, in *E. coli*, G-C transversions are the minor point mutation, and cytosine is preferentially inserted opposite NI, which leads to no mutation [24,54].

## 8. Spirodi(iminohydantoin) (5-Si and 8-Si)

**Figure 8:**
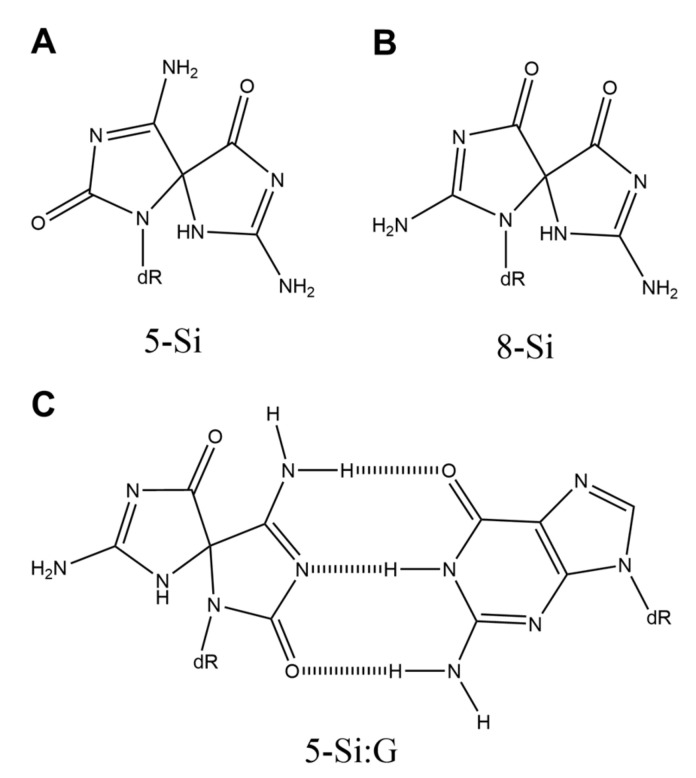
The structures of (**A**) 5-Si, (**B**) 8-Si, and (**C**) 5-Si:G.

Two diastereomers of spirodi(iminohydantoin) (5-Si and 8-Si) (Figure 8A,B) were first reported in 2015 and are produced by oxidation of guanine in the presence of NH_4_Cl [72]. Though base incorporation opposite 8-Si has not been analyzed, Klenow fragment exo^-^ incorporates adenine and guanine opposite 5-Si [72]. Since 5-Si mimics the hydrogen-bonding pattern of cytosine [72], a 5-Si:G base pair with three hydrogen bonds appears to be the structure shown in Figure 8C. On the other hand, in 2007, it was reported that the product having the same molecular weight as 5-Si and 8-Si is produced by oxidation of 8-oxoG with peroxynitrite [73]. Moreover, this unidentified product has caused G-C and G-T transversions in *E. coli*, and G-T transversions are the major point mutation [73].

## 9. Triazine and Unknown M+7 Product

**Figure 9:**
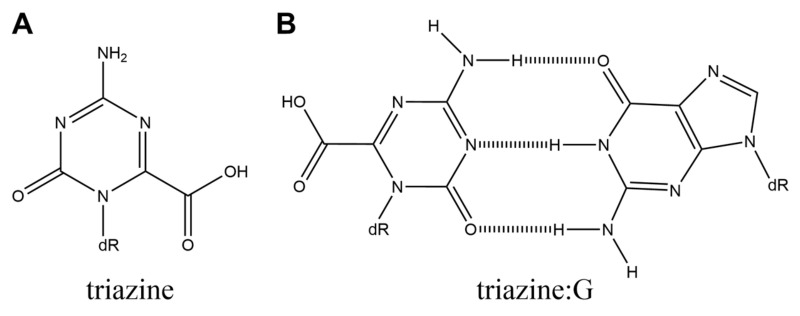
The structures of (**A**) triazine and (**B**) triazine:G.

Triazine (Figure 9A) is an oxidation product of guanine and 8-oxoG [74,75]. The product having the same molecular weight as triazine causes G-C and G-T transversions in *E. coli*, although the product has not been identified as triazine [73]. However, the thermal stability of DNA duplexes, base incorporation with polymerases, and calculations of base pairs have not been analyzed for triazine. Based on the structure of triazine, it can form a base pair with guanine (Figure 9B).

Moreover, the unidentified product having a mass corresponded to 7 amu (M+7) above that of guanine has been reported, and the M+7 product causes the greatest amounts of G-C transversions in addition to G-A transitions and G-T transversions [73].

## 10. Other Products by Peroxynitrite

In addition to NI, 8-nitroguanine is a nitration product of guanine [76]. Parabanic acid and *N*-nitro-dehydroguanidinohydantoin are produced by oxidation of 8-oxoG with peroxynitrite [77,78]. However, since these three products are unstable, researches on these products are unrealistic. 

On the other hand, 4-hydroxy-2,5-dioxo-imidazolidine-4-carboxylic acid (HICA) is a stable product of oxidative guanine damage [79,80,81], and the thermal stability of DNA duplexes containing HICA and base incorporation with polymerases opposite HICA have not been analyzed.

## 11. Alkylated Guanines

In the mutation spectrum for N7-methylguanine, N1-methylguanine, 8-methylguanine, and 1,*N*^2^-ethenoguanine, G-C transversions are observed [82]. However, opposite these alkylated guanines, cytosine is predominantly incorporated, and G-C transversions at these alkylated guanines are the minor point mutation [82].

## 12. 8,5′-Cyclo-2′-deoxyguanosine (cG)

8,5′-cyclo-2′-deoxyguanosine (cG) is the smallest tandem lesion generated by hydroxyl radical [83]. Guanine is the least incorporated base opposite cG by Klenow fragment exo^-^ [84] and *Sulfolobus solfolobus* P2 DNA polymerase B1 [85]. In addition, cytosine is preferentially incorporated by *Sulfolobus solfolobus* DNA polymerase IV [84,85], DNA polymerase IV [84], human and *Saccharomyces cerevisiae* DNA polymerase η [86], human DNA polymerase ι [87]. Human DNA polymerase κ [87] and *Saccharomyces cerevisiae* DNA polymerase ζ [87] incorporate cytosine and guanine. In *E. coli*, cG causes no mutations and G-A mutations [88,89]. From the above, cG is not a product of oxidative guanine damage that can cause G-C transversions.

## 13. Base Pairs Related to New Medicines Against Novel Coronavirus

The spread of the 2019 novel coronavirus (SARS-CoV-2) has caused a global pandemic. Many researchers are considering whether several existing antiviral agents against the influenza virus and others are effective in SARS-CoV-2 [90,91].

**Figure 10:**
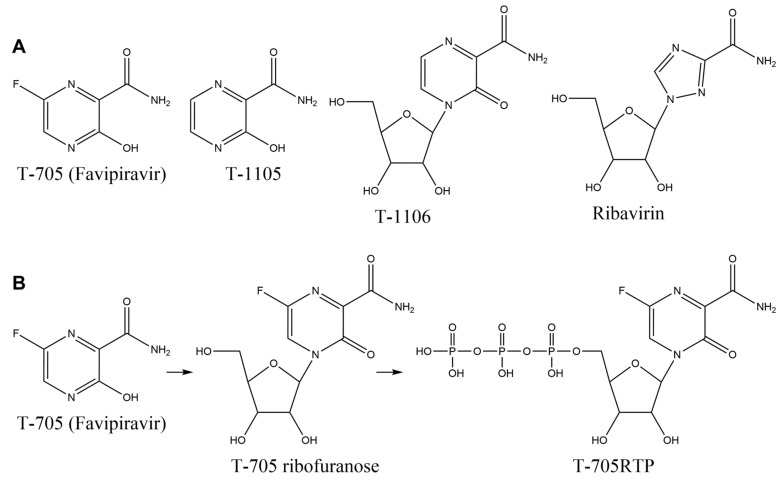
The existing agents against RdRp. (**A**) The structures of T-705, T-1105, T-1106, and ribavirin. (**B**) Converting T-705 to T-705RTP.

**Figure 11:**
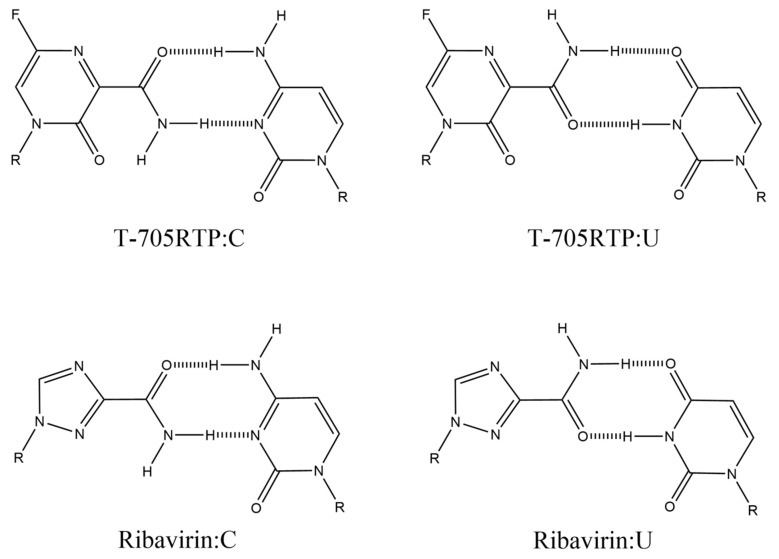
The structures of base pairs containing T-705 ribofuranose and ribavirin.

T-705 (favipiravir) (Figure 10A) is an antiviral drug that inhibits the RNA-dependent RNA polymerase (RdRp) of the influenza virus. T-705 eventually leads to its active form, T-705RTP (Figure 10B) [92,93]. T-705 has a broad-spectrum activity against other RNA viruses, such as the Ebola virus and others [94,95]. Since the catalytic region of the RdRp is widely conserved among the RNA viruses, T-705 is said to be effective against a wide range of RNA viruses. The inhibition of RdRp activity affects viral genomic replication, and forming base pairs is important for the viral suppression mechanism: T-705RTP has been proposed to form the base pairs with cytidine and uridine (Figure 11) [93].

The structure of T-1105 (Figure 10A) is similar to T-705, and T-1105 is also effective in the influenza virus [96]. T-1105 and T-1106 (Figure 10A) have potent antiviral activities against the dengue virus [97]. The base moiety has potential base pairs with cytidine and uridine like T-705 [97]. Via similar considerations, C-nucleoside analogs having amide show anti-influenza activity, and base pairs with cytidine and uridine have been proposed [98].

Ribavirin (Figure 10A) also shows antiviral activity against various RNA viruses, especially in the hepatitis C virus (HCV), and the structure having amide leads to suggested base pairs with cytidine and uridine (Figure 11) [99]. On the other hand, it has been reported that *N*^4−^hydroxycytidine, which acts as an analog of cytosine or uridine [100], is effective against several viruses [101].

Remdesivir leads to its active triphosphate and inhibits the RdRp of the Ebola virus [102] and others [103,104]. This drug, which is effective against a wide range of RNA viruses, also has antiviral activity against SARS-CoV-2 [105]. Because remdesivir mimics the structure of adenosine as a substrate, delayed chain termination of RNA synthesis using RdRps of SARS-CoV-2 [106], Nipah virus [107], and Ebola virus [108] show that remdesivir forms the base pair with uridine [105].

Thus, T-705 ribofuranose, T-1106, ribavirin, *N*^4−^hydroxycytidine, and remdesivir can act as natural nucleosides in the RNA replication, and the fact that drugs with different structures have effects on RdRps of several viruses suggests that the nucleic acid derivatives capable of forming base pairs have broad-spectrum antiviral activities. Therefore, some ribonucleoside analogs, which can form base pairs, are possible to be effective in SARS-CoV-2.

**Figure 12:**
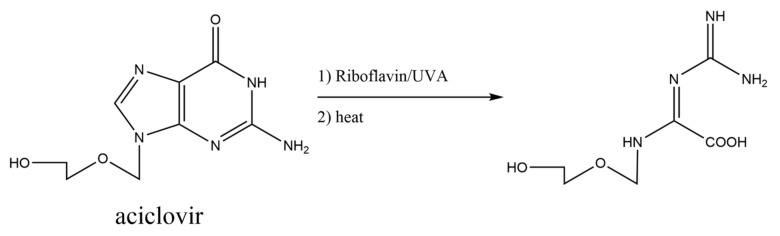
Proposed photosensitization adapted for aciclovir.

In the above sections, the base pairs containing oxidative guanine damages have been described. The reactions that produce DNA damages can also be adapted for the ribonucleoside analogs. In Figure 12, an example using aciclovir is shown. Taking into consideration that forming base pairs are important in the antiviral mechanisms against RNA viruses, some reaction products capable of forming hydrogen bonds with natural ribonucleotides may be drug candidates for SARS-CoV-2.

## 14. Conclusions and Future Studies

In this review, we have described the findings so far concerning products of oxidative guanine damage that can form base pairs with guanine. Iz forms mainly Iz:G base pairs. Oz, Gh/Ia, and Sp form base pairs, preferably with guanine in some cases. Gf, 2Ih, Ua, 5-Si, 8-Si, triazine, the M+7 product, and HICA have a few or no experiments on thermal stability, base incorporations, or mutations in *E.coli*. NI, alkylated guanines, and cG are not involved in G-C transversions. Recently, it has been reported that DNA base pairing is not controlled by DNA properties alone but by appropriateness for substrates in the polymerase active site [109]. Especially this discussion is important in consideration of a wide variety of bases incorporated by translesion synthesis polymerases. Conversely, when the preferentially incorporated base is independent of polymerases, forming base pairs is certainly important: We have collected information on typical generations of oligomer containing the damage, the *T*_m_ data, bases incorporated by polymerases except for translesion synthesis polymerases, and mutations in *E. coli* in Table 1. We note, however, that some lesions have not been analyzed with some polymerases, and that mutation spectrum analysis in human cells has not yet been performed. Further analyses are likely to reveal more detailed information about the mechanisms of G-C transversion.

In addition, to prevent the occurrence of mutations, DNA damages must be repaired [110]. Oxidative damages are usually repaired by base excision repair enzymes. For example, human NEIL1 and human NTH1 are active against Oz, but similar activities against Oz:C, Oz:G, and Oz:A are shown [111]. Considering the facts, at least human NEIL1 and human NTH1 do not depend on the stability of base pairs containing Oz. Therefore, in addition to the previously known results, it is necessary to newly measure the activity of various repair enzymes to determine whether the enzyme depends on the stability of the base pair or not.

On the other hand, for nucleotide excision repair, it is important for XPC-RAD23B to detect the bulge of the structure of DNA duplex. Previously, the stability of base pairs containing 5-formyluracil, which is oxidative damage to thymine, was correlated with nucleotide excision repair activity [112]. Therefore, in the future, it is necessary to study the correlation between the stability of base pairs containing oxidative guanine damage and nucleotide excision repair activity.

Lastly, in the Section 13 of this review, several antiviral drugs are described and forming base pairs inhibit the RdRp. Since some oxidative guanine damages can form base pairs, researchers may find drug candidates for SARS-CoV-2 when reactions producing oxidative guanine damage are adapted for the ribonucleoside analogs.

## Figures and Tables

**Table 1:** Oxidative guanine damages that mainly cause G-C transversions.

Damage	Typical Generations of Oligomer Containing the Damage ^1^	The Ease of Formation of Base Pairs ^3,4^
Iz	G/riboflavin, 366 nm [14,15]	*T*_m_: Iz:G > Iz:T > Iz:A > Iz:C [5]
		Pol I: Iz:G > Iz:A [14]
		Pol α: Iz:G ~ Iz:C > Iz:A [22]
		Pol β: Iz:G ~ Iz:C > Iz:A [22]
		*E. coli*: Iz:G > Iz:C [24]
Oz	Iz/heat [26]	*T*_m_: only Oz:G ^2^ [27]
		Kf exo^-^: only Oz:A [26]
		Kf exo^-^: Oz:A ~ Oz:G [22]
		Taq: Oz:A > Oz:G ~ Oz:C [26]
		Pol α: Oz:G > Oz:A [22]
		Pol β: Oz:G > Oz:A [22]
		Pol γ: Oz:G > Oz:A [22]
		Pol δ: only Oz:G [28]
		Pol ε: only Oz:G [22]
		*E. coli*: Oz:A > Oz:C [29]
Gf	Oz/heat [30]	Kf exo^-^: Gf:A > Gf:G > Gf:C [30]
Gh/Ia	8-oxoG/Na_2_IrCl_6_, 4 °C [35]	*T*_m_: Gh/Ia:A > Gh/Ia:C > Gh/Ia:G > Gh/Ia:T [36]
	8-oxoG/I_2_, KI, pH 5.7 [81]	Kf exo^-^: Gh/Ia:A > Gh/Ia:G [22,36]
		Kf exo^-^: Gh/Ia:G > Gh/Ia:A [36]
		RB69 exo^-^: Gh/Ia:A > Gh/Ia:G [43,44]
		Pol α: Gh/Ia:G ~ Gh/Ia:A [22]
		Pol β: Gh/Ia:G > Gh/Ia:A [22]
		Pol γ: Gh/Ia:A > Gh/Ia:G [22]
		Pol ε: Gh/Ia:A ~ Gh/Ia:G [22]
		RNA Pol II: Gh/Ia:A > Gh/Ia:G [45]
		SuperScript III: Gh/Ia:G > Gh/Ia:A [46]
		*E. coli*: Gh:G > Gh:A [47,48,54]
Sp	8-oxoG/Na_2_IrCl_6_, 50 °C [35]	*T*_m_: Sp:G > Sp:A > Sp:C > Sp:T [36]
	8-oxoG/I_2_, KI, pH 7.7 [81]	*T*_m_: Sp:C > Sp:G > Sp:A > Sp:T [36]
		*T*_m_: Sp1:G > Sp1:T > Sp1:A > Sp1:C [53]
		*T*_m_: Sp2:G ~ Sp2:T > Sp2:A > Sp2:C [53]
		*T*_m_: Sp2:G > Sp2:A > Sp2:T > Sp2:C [53]
		Kf exo^-^: Sp:A > Sp:G [36]
		Kf exo^-^: Sp:G > Sp:A [36]
		Klen Taq: Sp:G > Sp:A [56]
		RNA Pol II: Sp:A > Sp:G [45]
		SuperScript III: Sp:G > Sp:A [46]
		*E. coli*: Sp:G > Sp:A [47]
		*E. coli*: Sp1:A > Sp1:G [48,54]
		*E. coli*: Sp2:A ~ Sp2:G [48]
		*E. coli*: Sp2:G > Sp2:A [54]
		SOS-induced *E. coli*: Sp2:A > Sp2:G [54]
2Ih	G/Mn-TMPyP, KHSO_5_ [59]	*T*_m_: 2Ih:G > 2Ih:A > 2Ih:C > 2Ih:T [56]
	G/X-ray, ascorbate [62]	Kf exo^-^: 2Ih:G > 2Ih:A [56]
	G/Fe(II)-EDTA, H_2_O_2_, ascorbate [62]	Klen Taq: 2Ih:G > 2Ih:A [56]
	G/NiCR, KHSO_5_ [60]	
	G/(AcO)_2_Cu, ascorbate, H_2_O_2_ [61]	
Ua	oxalurate/ NaHCO_3_ [64]	*E. coli*: Ua:A > Ua:G > Ua:T [48]
		*E. coli*: Ua:A > Ua:G > Ua:T > Ua:C [54]
		*E.coli*: only Ua:A [64]
		SOS-induced *E. coli*: Ua:G > Ua:A > Ua:T [64]
NI	G/peroxynitrate [17]	*T*_m_: NI:G > NI:A > NI:C > NI:T [68]
	G/308 nm, NaHCO_3_, NaNO_2_, Na_2_S_2_O_8_ [67]	Pol α: NI:A > NI:G > NI:C [70]
		Pol β: NI:C > NI:A > NI:G [70]
		Kf exo^-^: NI:C > NI:A > NI:G [70]
		T7 RNA Pol: NI:C > NI:A > NI:G > NI:U [71]
		RNA Pol II: only NI:C [71]
		*E. coli*: NI:C > NI:A > NI:T > NI:G [24]
		*E. coli*: NI:C > NI:A > NI:G > NI:T [54]
		SOS-induced *E. coli*: NI:C > NI:A > NI:T > NI:G [54]
5-Si	G/riboflavin, NH_4_Cl, 350 nm [72]	Kf exo^-^: 5-Si:A > 5-Si:G > 5-Si:C ~ 5-Si:T [72]
	G/rose bengal, NH_4_Cl, 350 nm [72]	(*E. coli*: ?:A > ?:T > ?:G > ?:C)^5^ [73]
	G/Na_2_IrCl_6_, NH_4_Cl [72]	(SOS-induced *E. coli*: ?:A > ?:G > ?:T > ?:C)^5^ [73]
	(8-oxoG/peroxynitrate) [73]	
triazine	(8-oxoG/peroxynitrate) [73]	(*E. coli*: ?:G > ?:A > ?:T > ?:C)^5^ [73]
		(SOS-induced *E. coli*: ?:A > ?:G > ?:T > ?:C)^5^ [73]
M+7	(8-oxoG/peroxynitrate) [73]	(*E. coli*: ?:G > ?:T > ?:A > ?:C)^5^ [73]
cG		Kf exo^-^: cG:C > cG:T > cG:A ~ cG:G [84]
		Pol B1: cG:C > cG:A > cG:T > cG:G [85]
		*E. coli*: cG:C > cG:T [88,89]

^1^ All detection methods are HPLC. ^2^ The *T*_m_ values of Oz:C, Oz:T, or Oz:A are not determined and below 40 °C. ^3^ This table does not contain the data of base incorporations by translesion synthesis polymerases. ^4^ “Damage:Base 1 > Damage:Base 2” means that the *T*_m_ value of Damage:Base 1 is higher than that of Damage:Base 2, or that Base 1 is incorporated more preferentially than Base 2 by polymerases or in *E. coli*. “Damage:Base 1 ~ Damage:Base 2” means that the *T*_m_ value of Damage:Base 1 is almost the same as that of Damage:Base 2, or that Base 1 and Base 2 are incorporated to the same degree by polymerases or in *E. coli*. “Pol” is an abbreviation of “Polymerase”. “Kf” is an abbreviation of “Klenow fragment”. ^5^ The unidentified product “?” having the same mass as each damage is used.

## Abbreviations

Iz	2,5-diamino-4*H*-imidazol-4-one
Oz	2,2,4-triamino-5(2*H*)-oxazolone
Gf	guanidinoformimine
Gh	guanidinohydantoin
Ia	iminoallantoin
Sp	spiroiminodihydantoin
2Ih	5-carboxamido-5-formamido-2-iminohydantoin
Ua	urea
8-oxoG	8-oxoguanine
E. coli	Escherichia coli
Klenow fragment exo^-^	the large fragment of *Escherichia coli* DNA polymerase I exonuclease minus
dR	deoxyribose
NI	5-guanidino-4-nitroimidazole
Si	spirodi(iminohydantoin)
HICA	4-hydroxy-2,5-dioxo-imidazolidine-4-carboxylic acid
cG	8,5′-cyclo-2′-deoxyguanosine
SARS-CoV-2	2019 novel coronavirus
RdRp	RNA-dependent RNA polymerase
HCV	hepatitis C virus
R	ribose
